# The effects of sleep restriction and time of day on food-specific impulsivity, approach-avoidance bias and delay discounting

**DOI:** 10.1080/21642850.2025.2520838

**Published:** 2025-06-20

**Authors:** Naomi Kakoschke, David L. Dickinson, Sean P.A. Drummond

**Affiliations:** a Human Health, Health & Biosecurity, CSIRO, Adelaide, Australia; b Economics and CERPA, Appalachian State University, Boone, NC, USA; c Economic Science Institute (ESI), Chapman University, Orange, CA, USA; d Institute of Labor Economics (IZA), Bonn, Germany; e School of Psychological Sciences, Monash University, Clayton, Australia

**Keywords:** Sleep restriction, circadian timing, approach-avoidance bias, delay discounting, impulsivity, food choice

## Abstract

**Background:**

Insufficient sleep and circadian timing are both linked with obesity, primarily via unhealthy food choice, yet the cognitive mechanisms underpinning such relationships remain unclear.

**Methods:**

Across two studies, we implemented an ecologically valid within-subjects at-home protocol. Study 1 (*n* = 118) involved a within-subjects examination of how sleep restriction (SR) versus well-rested (WR) sleep levels affect choices in a food-based approach-avoidance task (AAT) and go–no/go (GNG) task, a food liking task, a food-choice task, a psychomotor vigilance task (PVT), and a monetary choice task. Study 2 (*n* = 119) involved examining choices in the same set of tasks administered once in the afternoon (4pm) and once during the night (4am), which leveraged circadian influences on sleepiness and cognitive function.

**Results:**

During the night, participants indicated steeper discounting rates relative to the afternoon. Furthermore, such rates predicted higher liking of high-calorie food choices regardless of time of day and when sleep restricted. Approach bias for low-calorie food interacted with the night condition in predicting both low- and high-calorie food choices.

**Conclusion:**

Both delay discounting and approach bias may be important cognitive mechanisms predicting food liking and choice under sleep restricted and altered circadian timing conditions. Further research should replicate such results using real rewards.

Insufficient sleep (<7 h/night) (National Institutes of Health, 2022) is a significant global health concern (Ford et al., 2015; Hafner et al., 2017; Hirshkowitz et al., 2015; Watson et al., 2015). Over 80 million U.S. adults (≈ 35%) suffer from insufficient sleep (Liu, 2016), which is similar to the adult obesity rate (39%) (Hales et al., 2017). Furthermore, homeostatic pressure and circadian timing impact the physiological ability to sleep, also known as sleep propensity according to the 2-process model of sleep regulation (Borbély, 1982). Disruptions to either of these processes will impair sleep quality and quantity. Thus, the combined public health concerns of insufficient sleep and obesity have widespread impact and are considered key risk factors for chronic disease (Liu Yong et al., 2016). There is a recognized link between poor sleep and obesity, but studies have argued for causation both ways (Beebe et al., 2007; Campanini et al., 2017; Chaput, 2014; Garaulet et al., 2011). In other words, poor sleep and obesity are concomitant, but the direction is difficult to disentangle (Castillo et al., 2017).

A critical factor driving obesity is food choices, given that diet quality is a key determinant of health. Indeed, modifiable health behaviors are one of the pathways by which sleep and circadian timing impact chronic disease risk (Baron & Culnan, 2019). Similarly, eating behavior is also influenced by circadian timing and biological rhythms, with the lowest drive to eat occurring in the morning and the highest in the evening. Research shows that circadian disruption impacts health behaviors including dietary intake (Brondel et al., 2010). There are many mechanisms through which sleep loss and/or circadian disruption impacts dietary intake. For example, both sleep restriction and circadian misalignment negatively affect levels of ghrelin and leptin, glucose tolerance, and insulin sensitivity. One study concluded that short sleep duration affected food choice through its impact on appetite hormones, specifically, ghrelin, which relates to hunger and food intake, and leptin, which regulates energy balance by suppressing food intake (Klok et al., 2007). Relatedly, a longitudinal study of over 1000 participants found that short sleep duration was associated with higher ghrelin and lower leptin levels (Taheri et al., 2004). Short sleep duration may also decrease glucose tolerance and insulin sensitivity (Spiegel et al., 2005). However, a randomized cross-over study of healthy men found that SR did not affect ghrelin and leptin concentrations, although it did lower physical activity levels (Schmid et al., 2009). Hogenkamp et al. (2013) conducted a cross-over study using total sleep deprivation (TSD) and showed that overeating after TSD was restricted to mornings because of both hedonic (preference based) and homeostatic (biological regulation based) factors. Other research has documented elevated levels of ghrelin during one’s circadian evening (Qian et al., 2019), which may promote overeating or unhealthier food choices at night (Chaput et al., 2023).

This present study focuses on other mechanisms through which sleep disruption can affect dietary intake, specifically, changes in neurocognitive factors including attention, executive function, and delay discounting. Such mechanisms have been previously noted (see review in Goel et al., 2009), albeit few studies examined research in applied settings (Baron & Culnan, 2019). Thus, establishing a solid understanding of the channels through which sleep may impact dietary choices in applied settings is vital. To our knowledge, research to date has not comprehensively examined key cognitive processes underlying food choice together nor under altered sleep. Given that numerous confounds are present in naturally occurring data, experimental manipulation of sleep and laboratory decision paradigms offer advantages regarding hypothesis testing. Obesity is also not the only risk factor for insufficient sleep that may manifest in naturally occurring data (Horne, 2011). This may unfortunately divert attention away from a systematic study of the behavioral impacts of insufficient sleep that may hold clues to understanding an important input into the obesity equation, namely, dietary choice.

Dietary choice, in turn, is influenced by underlying cognitive processes. Research examining the cognitive components of unhealthy eating behaviors has demonstrated that impulsivity and cognitive biases impact food choice, namely, processes that reflect less deliberative decision-making (Kakoschke et al., 2017b; Kakoschke et al., 2019). Impulse control, decision bias, and present-bias time preferences are all domains of decision-making vulnerable to the negative impact of insufficient sleep. Studies on impulse control have consistently shown that 1–2 nights of total sleep deprivation worsens performance (i.e. increases inhibition errors) on a Go/No–Go task relative to normal sleep (Almklov et al., 2015; Chuah et al., 2006; Drummond et al., 2006). More closely related to our project, emerging evidence suggests that 1 night of total sleep deprivation (TSD) impairs performance on a food cue specific Go/No–Go task (Cedernaes et al., 2014), albeit the findings were limited in power and generalizability due to use of a small (*N* = 14) sample of young men. Only a couple of studies have examined the effect of insufficient sleep on impulse control. These authors reported impaired performance on a Go/No–Go task after 4 nights (Demos et al., 2016) but not 1 night of SR (Rossa et al., 2014). Research is needed into the food-specific mechanisms of impulse control and decision-making following insufficient sleep.

Sleep loss also affects cognitive functions involved in decision-making such as delay discounting, a critical construct in behavioral economics. Delay discounting refers to the idea that rewards subjectively decrease in value (i.e. become devalued) as a function of delayed receipt (Madden & Bickel, 2010) whereby people make choices in line with immediate gratification rather than improved health in the future. The idea that people prefer smaller, immediate rewards over larger, delayed rewards is proposed to be a trans-diagnostic behavioral process (Bickel et al., 2019). Delay discounting is typically measured via monetary choice tasks or questionnaires to assess how quickly rewards reduce in value. While delay discounting paradigms often use monetary rewards, they can successfully predict BMI (Epstein et al., 2014) and weight gain across time among adults with obesity (Kishinevsky et al., 2012; Weller et al., 2008). A meta-analysis on the relationship between delay discounting and obesity indicated an overall medium effect size across both food and monetary rewards (*d* = 0.43), albeit a larger effect size for food rewards (Amlung et al., 2016). Furthermore, some studies have shown that food, but not monetary rewards predicted increased body fat percentage in healthy adults (Rasmussen et al., 2010). In the sleep domain, studies on delay discounting have reported mixed findings. Specifically, sleep loss (partial or total) has been linked to a preference for smaller, immediate rewards, over larger delayed rewards in some studies (Curtis et al., 2018; Reynolds & Schiffbauer, 2004), but not others (Acheson et al., 2007; Demos et al., 2016; Libedinsky et al., 2013; Martin et al., 2015).

Differential results across these delay discounting studies may relate to methodological differences (e.g. observational self-reported sleep levels versus experimentally assigned sleep restriction, time discounting related to money versus effort, hypothetical versus real rewards). Our methodology is most like that in Demos et al. (2016), who used an at-home partial sleep restriction protocol with the same money-based time discounting task. They reported no changes in estimated time discount rates with sleep restriction, but our Study 1 with similar methodology benefits from a much larger sleep restriction sample (*n* = 118 versus *n* = 37), somewhat less restrictive participant conditions (their sample excluded left-handed adults, tobacco users, those taking part in a weight loss program), a longer at-home sleep treatment period (7 nights versus their 4 nights), and real monetary incentives (Demos et al.’s were hypothetical). As such, our study offer valuable new evidence on how incentivized monetary-based delay discounting responds to experimentally assigned sleep restriction. Overall, more research is needed into the effects of sleep loss on delay discounting and the effects on food-related decision-making, and our study also adds to this research by contributing novel data on the effects of adverse time-of-day and monetary delay discounting in our Study 2.

Approach-avoidance bias is a key cognitive component of decision-making implicated in dietary choice. Approach biases for high-calorie foods are *automatic* action responses resulting from associative links between food cues (e.g. the sight of chocolate) and the rewarding experience of eating such foods (Kemps et al., 2013). Once established, approach biases are involved in the development of unhealthy habits (e.g. nibbling on chocolate snacks; Kakoschke et al., 2015) that can overrule conscious intentions (e.g. nibbling on chocolate snacks while on a diet) (Watson et al., 2012). Studies have consistently demonstrated faster approach responses toward high-calorie foods (Brignell et al., 2009; Kemps & Tiggemann, 2015). Crucially, approach biases for high-calorie foods are stronger among overweight individuals (Kakoschke et al., 2017a). These biases are linked with increased food intake and weight gain (Nederkoorn et al., 2010). These cognitive mechanisms may importantly contribute to explaining unhealthy dietary choices that underpin the current obesity epidemic.

Chronic insufficient sleep or sleep restriction (SR) is more commonly experienced than extreme sleep loss or TSD. Yet, most lab research on sleep and decision-making has involved extreme sleep loss in a sleep lab where external validity is lower. Complementary studies examining sleep loss under more ecologically valid conditions are therefore critical in efforts to document the robustness of sleep-lab findings (e.g. Dickinson & McElroy, 2017) replicates those of Anderson & Dickinson (2010). However, relatively few studies have examined the impact of *partial* sleep loss on decision-making processes (Alhola & Polo-Kantola, 2007). Early at-home sleep protocols involved small samples with only a single night of sleep manipulation (Sadeh et al., 2011) or imposed stringent SR conditions over the course of a week that resulted in nightly sleep of less than 5 h, on average (Van Dongen et al., 2004). In fact, such levels of at-home SR led some authors to argue against at-home SR protocols altogether (Van Dongen et al., 2004). Surveys report higher proportions of adults with nightly sleep between 6 and 7 h compared to the proportion sleep less than 6 h per night (Adams et al., 2017; Hafner et al., 2017), such that a study of less extreme levels of insufficient sleep has broader applications. The prevalence of these milder levels of insufficient sleep in modern societies highlights the relevance of examining how adverse sleep-related cognitive states may impact these critical components of food-related decision-making.

## The current study

The primary aim of the current study was to determine the impact of insufficient sleep and suboptimal time-of-day on cognitive processes relevant for dietary choice (e.g. time discounting rates, inhibitory control, and approach-avoidance bias) alongside food liking and choices for both low- and high-calorie foods. We hypothesized reduced deliberative processing under SR and adverse time-of-day (TOD) as indicated via poorer inhibitory control for high-calorie foods, stronger approach biases for high-calorie foods, and higher time discounting rates alongside higher liking ratings and choices for high-calorie foods. An exploratory aim was to determine whether these cognitive processes moderated the impact of treatment condition on food liking and choices. We hypothesized that stronger approach poorer inhibitory control for high-calorie foods, stronger approach biases for high-calorie foods, and higher time discounting rates would predict higher liking ratings and choices for high-calorie foods in the sleep restricted and suboptimal TOD conditions.

## STUDY 1: sleep restriction effects food-relevant cognitive tasks

Study 1 examined the impact of sleep restriction on inhibitory control, approach-avoidance bias and money-based delay discounting in a preregistered cross-over design experiment.

### Methods

Preregistrations relevant to Study 1 were done using the Open Science Framework (registrations https://doi.org/10.17605/OSF.IO/NSPRK for the Study 1 sleep protocol, and https://doi.org/10.17605/OSF.IO/BKH5J for the decision tasks methods). The study received ethics approvals for human subjects research by the Institutional Review Board at Appalachian State University (IRB# for study approval IRB-19-0188), and all participants gave voluntary informed consent to participate.

### Participants

Participants were prescreened for eligibility from a mid-sized U.S. University population. Exclusion criteria included: older than 40 years of age (or younger than 18), self-reported or suspected sleep or eating disorder or dietary restriction, extreme diurnal preferences as validated by the reduced Morningness-Eveningness Survey: (Adan & Almirall, 1991), significant risk of major depressive or generalized anxiety disorders as validated by the PHQ-2 (Kroenke et al., 2003) and the GAD-7 (Spitzer et al., 2006) instruments, respectively. While not exclusion criteria, participants also reported sex, race, and ethnicity in the prescreening survey, and once enrolled they also self-reported height and weight during the initial laboratory session such we have body mass index (BMI) measures for Study 1.

Our preregistration plans targeted a total enrollment of *n* = 160 participants, which were exceeded slightly due to available budget funds. In total, we enrolled *n* = 176 participants into the study (treatment condition: *n* = 137; control condition: *n* = 39), and a total of *n* = 155 participants completed the study and were included in our analysis (*n* = 118 treatment, *n* = 37 control). The preregistered control condition sample size was smaller than for the treatment condition given our main intent was to use control participants as a comparison group in assessing validity of the sleep level manipulation (i.e. treatment condition) protocol. Ex ante power analysis indicated that our minimum planned treatment group sample size of *n* = 100 would have power = 0.80 to detect small-medium-sized effects of sleep restriction on decision-making at the standard 0.05 alpha error probability across a variety of potential analytical approaches (e.g. ANOVA, linear multiple-regression) for the within-subjects design.

### Design and experimental manipulation

**Figure 1.**
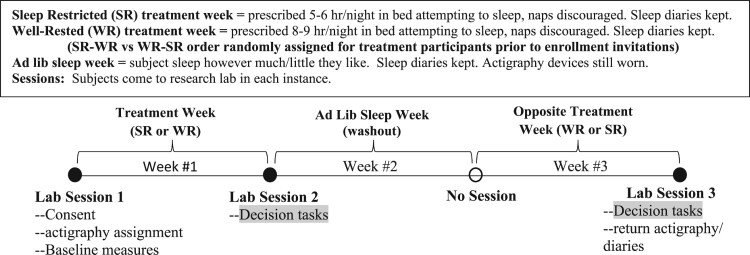
Study 1 cross-over design timeline (enrolled Treatment group participants).

The study protocol was a within-subject cross-over design sleep manipulation. The total duration was 3 weeks whereby weeks 1 and 3 involved exogenously assigned nightly sleep levels verified using objective actigraphy measures of sleep duration. The well-rested (WR) treatment assigned participants to attempt 8–9 h of sleep each night of that week, and the sleep-restricted (SR) treatment assigned participants to attempt 5–6 h of sleep each night of that treatment week. Week 2 of the protocol was an ad-lib sleep level week for all participants, and treatment condition participants were randomly assigned to an WR-SR or SR-WR order of the Treatment Weeks 1 and 3, while control condition participants were assigned WR-WR for Weeks 1 and 3. Importantly, at the end of Weeks 1 and 3 (i.e. after a full week of treated sleep levels), participants completed the in-lab cognitive tasks we report in this study. Thus, treatment participant completed the decision tasks both in a WR and SR state (in randomized order), while control participants completed the decisions tasks twice in a WR state. Figure 1 shows the protocol design and timeline for those randomly assigned as Treatment group participants. The design is identical for Control Group Participants except that both Weeks #1 and #3 are assigned as WR sleep treatment weeks. This approach has previously been successful at achieving moderate levels of at-home SR (5.5 ± 0.43 h) without any reported adverse events (Dickinson et al., 2017). These levels of SR are more in-line with levels reported in a larger segment of the population (Liu, 2016) and can be examined in a relatively safe at-home protocol when certain risk management features are used. For example, our protocol allows consumption of caffeine or use of other compensatory strategies. Of course, this approach sacrifices some experimental control but enhances ecological validity while offering a measure of risk management in the protocol (Dickinson et al., 2017).

### Procedure

All eligible participants were randomly assigned to the treatment or control condition prior to recruitment invitations. Enrolled participants had three laboratory sessions across the 3-week protocol. Day 1 was an initial laboratory session used to collect some baseline data on all participants (e.g. measures of cognitive and eating control tendencies, as well as body mass index information), and to instruct participants on use of the actigraphy sleep-tracking device (Actiwatch Spectrum Plus) and sleep diaries that were used to score self-reported sleep data. Sleep-tracking actigraphy devices were *not* consumer-oriented such that participants could not view their own sleep data during the study, and thus, they were not provided with feedback during the protocol. Importantly, the sleep levels in our study were experienced in one’s at-home naturalistic environment. After the end of Weeks 1 and 3 (i.e. after a full week of treated sleep levels), participants completed the incentivized in-laboratory cognitive tasks, namely, the approach-avoidance task, the Go/No–Go task, and the Monetary Choice task. Other tasks related to food choice are also reported, and participants were administered a psychomotor vigilance task and subjective sleepiness questionnaire to both objectively and subjectively assess alertness and sleepiness after each treatment week. Actigraphy data were scored after the participant had completed the study to objectively assess adherence to the sleep protocol.

### Measures

*Approach-Avoidance Task (AAT):* To measure approach-avoidance bias for food, we used a food-specific version of the AAT (Basanovic et al., 2023; Chen & Bargh, 1999). Participants were instructed to use their mouse to pull towards or push away from themselves the food images based on presentation format (i.e. portrait or landscape, with push/pull of portrait/landscape images counterbalanced). Image size will increase when pulling (simulating approach) and decrease when pushing (simulating avoidance) images. High-calorie foods appeared in pull-format on 50% of the trials, in push-format on the other 50%, and vice versa for low-calorie food images. So, participants avoided (pushed away) and approached (pulled toward) an equal proportion of high- and low-calorie images (i.e. 50/50). Images of 20 high-calorie and 20 low-calorie foods each shown twice resulting in a total of 80 trials (6 min). Images were from a validated database of food images (Blechert et al., 2019)and selected based on calorie-content of the pictured food. Several practice trials helped familiarize participants with the mouse-directed approach-avoidance technique prior to the consequential trials that were incentivized. The primary outcomes were AAT difference scores (median latency of correct responses for push trials – median latency of correct responses for pull trials), which were calculated separately for healthy and unhealthy foods. Positive scores indicated an approach bias (pushing slower than pulling), while negative scores indicated an avoidance bias (pulling slower than pushing). A monetary incentive was provided for overall AAT task accuracy on non-practice trials.

*Go/No–Go Task (GNG):* A food-specific version of the GNG task was used to assess inhibitory control for food (Teslovich et al., 2014). Target (‘go') stimuli appeared on 75% of trials to develop a prepotent response pattern, while non-target (‘no-go') stimuli appeared on 25% of trials. The task was administered across four blocks with each cue type (food or toys) serving as both a target and a non-target. Due to potential individual variability in consistently identifying foods as high- or low-calorie, food cues were grouped by calorie level and presented in separate runs. Within a given run, the food cues were all high- or all low-calorie. Stimuli were presented for 500 ms with an intertrial interval of 2000–4000 ms. The order of the four conditions of high-calorie food ‘go' with toy ‘no-go', low-calorie food ‘go' with toy ‘no-go', toy ‘go' with high-calorie food ‘no-go', and toy ‘go' with low-calorie food ‘no-go' were counter balanced across participants. The primary outcome measure for the GNG task was the number of commission errors for high-calorie food, namely, the number of incorrect responses (i.e. key presses) on no–go trials. Participants completed a three-minute practice session to ensure they understood and could follow the instructions, followed by *n* = 192 real trials (∼12 min). A monetary incentive was given for overall GNG task accuracy on the non-practice trials.

*Monetary Choice Questionnaire (MCQ):* The MCQ assesses preferences for smaller, immediate rewards over larger, delayed rewards using a 27-item self-administered questionnaire (Kirby et al., 1999). For each item, the participant was asked to choose between a smaller, immediate monetary reward and a larger, delayed monetary reward (e.g. Would you prefer $54 today, or $55 in 117 days?). The task protocol was scored by calculating where the respondent’s answers placed them amid reference discounting curves with placement amid steeper curves indicating steeper time discounting (Kaplan et al., 2016). The MCQ has been shown to be temporally stable and is highly correlated (*r* = 0.82) with computerized experimental methods. Following previous established methods of incentivization, subjects were informed that one of the 27 trials would be drawn at random, and they each faced a 1-in-6 chance of receiving their chosen payoff via an Amazon Gift code (Kirby et al., 1999). Choices from the MCQ trials are used to construct a participant’s discount rate, where higher discount rate implies a greater preference for immediate payoffs, and this discount rate can be separately specified for trials that include low, medium, and high dollar amount choices. Although the focus of this study was how SR impacts dietary choice relevant decision-making, outcomes from the MCQ are relevant towards a more general understanding of how SR impact time discounting.

*Food Liking:* Participants rated the tastiness of a total of 40 food images, of which 20 were high-calorie and 20 were low-calorie as per previous research (Benjamins et al., 2021). The images were taken from a validated and standardized image set, namely, the Food Pics Database (Blechert et al., 2019). Images were presented in a semi randomized order with the constraint that no more than three consecutive stimuli of the same category (low- or high-calorie) were presented in a row. Images were presented in the center of the screen on a black background with an intertrial interval of 300 ms. On each trial, participants were instructed to indicate how much they liked the taste of the presented food at the current moment on a 9-point scale ranging from 1 = ‘Very untasty’ to 9 = ‘Very tasty’. The mean liking rating for low- and high-calorie food items was calculated.

*Food Choice:* Participants were given 15 s to select eight snack foods that they would hypothetically like to consume from a grid of 16 food images (eight healthy; eight unhealthy) displayed on a touchscreen (Veling et al., 2013). The outcome was the number of high-calorie foods selected out of the available choices.

*Protocol Validity Measures:* Participants were administered the Karolinska Subjective Sleepiness (KSS) scale (Akerstedt & Gillberg, 1990) prior to the decision tasks, and a Psychomotor Vigilance Task (PVT; Thomann et al., 2014; Van Dongen & Dinges, 2005) at the end of the decision tasks. The PVT was used to assess objective sleep treatment impact and we administered a validated shorter duration than is typical, namely, 5 instead of 10 min (Roach et al., 2006) due to time constraints.

#### Data analysis

Participant responses on the AAT, GNG, and MCQ tasks were extracted and used to construct standard outcome measures that averaged across all comparable trials in the task. Multivariate regression models were estimated to examine key predictors of task outcome measures. The key independent measure of interest was a binary indicator variable *SR* = 1 if decisions followed the SR treatment week, and *SR* = 0 if they followed a WR treatment week. Control variables were included in the analysis to account for age (in years), sex (Female = 1), and minority status ( = 0 if White and Caucasian), validated morningness-eveningness score (MES: obtained from the prescreening survey), BMI (height and weight reported during the Session 1 lab visit of Study 1), and repeat administration of the task (given that each participant completed all tasks at the end of both Weeks 1 and 3 of the protocol). The models estimated in the main text (unless otherwise stated) were random effects generalized least squared regressions that account for multiple observations per participant (*n* = 2 observations per participant in the repeated measures data set).

Finally, because not all enrolled participants completed the protocol, we also conducted sensitivity analysis that accounted for potential sample selection by using the inverse probability weight correction (IPW) in a weighted regression (see Appendix Table A1 for selection equation estimation results used to generate the probability weights). In general, we indicate any differences found in the IPW-corrected sensitivity analysis in the main text, but we relegate those results tables to the Appendix. These weighted regressions also included robust standard errors clustered on the participant.

## Study 1 results

### Participant characteristics

Table 1 shows the sample characteristics Study 1 participants separated by treatment versus control participants.

**Table 1.** Sample characteristics for Study 1 (by group allocation).

	Study 1: treatment	Study 1: control
Characteristic	Mean (std. dev)	Mean (std. dev.)
Age (in years)	19.88 (1.97)	20.11 (3.93)
Female (%)	0.55 (.50)	0.62 (.49)
Minority (%)	0.11 (.31)	0.19 (.40)
BMI	25.18 (5.50)	25.07 (3.90)
MES	13.60 (3.04)	13.76 (3.28)
Optimal hours nightly sleep	8.06 (1.00)	8.54 (.80)
Total complete participants (*n*)	118	37

Note: ME, morningness-eveningness score. BMI, body mass index.

#### Protocol validity

**Figure 2.**
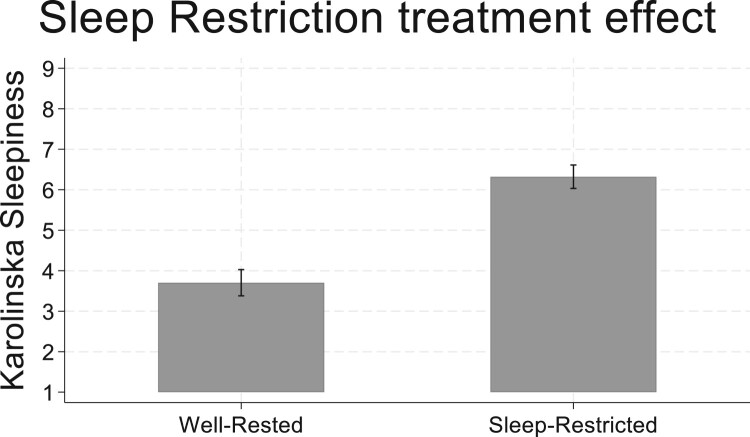
Study 1: Sleep Restriction treatment effect on sleepiness. Mean subjective sleepiness ratings with 95% confidence interval.

Protocol validity was determined via self-reported sleepiness ratings on the KSS and objective performance on the PVT. Focusing on the treatment participants, the SR treatment week produced significantly higher self-reported sleepiness ratings, relative to the WR treatment week (see Figure 2 – *Mann–Whitney non-parametric test: z* *=* *−10.224; p* *<* *.001*)*.* For the objective PVT outcomes, given the count-nature of the PVT lapse data, we estimated Poisson regressions and report that the SR condition significantly increased PVT lapses (*p* < .05; 1-tailed test given preregistered hypotheses). Random effect generalized least squares estimates were more appropriate for analysis of the ‘inverse of the mean lapse response times' measure commonly used as another measure in the PVT task (random effects take into account the panel nature of the data). Here, we found no treatment effects on the inverse mean lapse response times (see Table 2). While we focus on the treatment participants in our task outcomes analysis, we further document the Study 1 protocol validity by noting that self-reported sleepiness did not differ across the two WR treatment weeks in the smaller sample of control participants (*p* > .05 for the Mann–Whitney test of differences).

**Table 2.** Psychomotor vigilance task (PVT) outcomes (treatment validation) – Study 1.

	Study 1	
	(1)	(2)
Variables	PVT: # lapses	PVT: Inverse of the Mean Lapse RT
SR (=1)	0.284*	−0.018
	(0.168)	(0.020)
Age	−0.088	0.001
	(0.094)	(0.005)
Female (=1)	−0.330	0.003
	(0.342)	(0.025)
Minority (=1)	−0.109	−0.010
	(0.325)	(0.022)
BMI	0.030*	0.000
	(0.015)	(0.001)
MES	0.001	−0.000
	(0.041)	(0.002)
Repeat Admin (=1)	0.433**	0.032
	(0.147)	(0.017)
Constant	1.013	0.002
	(1.911)	(0.116)
Observations	234	121
*R*-squared		0.0299
Chi-Squared	25.08**	

Note: SR, sleep restricted, MES, morningness-eveningness score. BMI, body mass index.

*p < .05, **p < .01 for the 1-tailed test of a preregistered hypothesis (otherwise, 2-tailed test *p*-value reported). Coefficient estimates shown with robust standard errors clustered on the participant in parenthesis (*n* = 2 observations per participant). Model (1) is a Poisson regression given the dependent variable (# lapses) involves count data.

### Main preregistered hypotheses tests

The primary analyses were aimed at examining the preregistered hypotheses focused on the coefficient estimates for the SR variable in Study 1. To help contextualize our analyses, mean values of the key outcome measures by treatment condition, their standard deviations, and between-condition test results with effect sizes are shown in Table 3.

**Table 3.** Study 1 outcome measures descriptive statistics.

	Well-rested (WR)	Sleep restricted (SR)	Between-condition difference test *p-value* (effect size)
*N* (participants)	118 (50.0%)	118 (50.0%)	
Approach bias – high-calorie foods	−3.335 (74.671)	12.263 (132.563)	0.27 (−0.15)
Approach bias – low-calorie foods	0.805 (71.941)	2.691 (81.124)	0.85 (−0.02)
Go-NoGo commission errors – high-calorie foods	0.281 (0.205)	0.240 (0.178)	0.11 (0.21)
Go-NoGo commission errors – low-calorie foods	0.302 (0.198)	0.266 (0.175)	0.14 (0.19)
Overall discount rate (*k*-value)	0.016 (0.020)	0.017 (0.023)	0.74 (−0.04)
Small $ values discount rate (*k*-value)	0.029 (0.038)	0.032 (0.040)	0.57 (−0.07)
Medium $ values discount rate (*k*-value)	0.017 (0.022)	0.016 (0.022)	0.78 (0.04)
Large $ values discount rate (*k*-value)	0.011 (0.016)	0.012 (0.018)	0.57 (−0.07)

Notes: means shown with standard deviations in parenthesis. Approach Bias = 0 value represents no bias. For Go-NoGo, the commission errors value is the proportion of NoGo trials with an error. One-sample *t*-tests of the Approach Bias values for each treatment indicate Approach Bias scores for both high and low-calorie foods are not significantly different from zero in either the SR or WR condition (*p* > .10 in all instances for the two-tailed test). For the Between-Condition tests reported above, we ran two sample *t*-tests and report two-tailed *p-*values with Cohen’s *d* effect sizes in parenthesis.

### Food approach bias, commission errors, and discount rates

There were no significant effects of the sleep restriction treatment, relative to a more well-rested state, on approach-avoidance biases or commission errors for low- or high-calorie food, or on discount rates in Study (see Table 4). Robustness checks were conducted to account for potential sample selection among those who completed the study, and similar null results were found overall (see Appendix A, Table A3).

**Table 4.** Sleep restriction treatment condition effects on: approach bias scores, commission errors, and delay discounting rates – study 1.

	Approach-avoidance		Go–No Go		Monetary choice questionnaire			
	Approach bias		Commission errors		Discounting rate			
Variables	HC food	LC food	HC food	LC food	All $	Small $	Med $	Large $
SR (=1)	15.597	1.886	−0.040	−0.036	0.001	0.003	−0.001	0.001
	(12.460)	(10.147)	(0.025)	(0.025)	(0.002)	(0.003)	(0.002)	(0.002)
Age	7.369	−3.622	0.003	0.000	−0.001	−0.001	−0.001	−0.001
	(7.238)	(3.472)	(0.005)	(0.006)	(0.001)	(0.001)	(0.001)	(0.001)
Female (=1)	7.281	5.935	−0.033	0.002	−0.007	−0.009	−0.009*	−0.005
	(13.612)	(10.227)	(0.025)	(0.024)	(0.004)	(0.007)	(0.004)	(0.003)
Minority (=1)	57.225	21.670*	−0.034	0.041	−0.001	0.008	−0.003	−0.001
	(29.993)	(10.070)	(0.046)	(0.036)	(0.004)	(0.010)	(0.003)	(0.003)
BMI	−0.891	0.456	0.001	−0.000	0.000	0.000	0.000	0.000
	(1.008)	(0.875)	(0.002)	(0.002)	(0.000)	(0.001)	(0.000)	(0.000)
MES	−2.934	−1.759	0.001	0.003	0.000	0.001	0.001	0.000
	(2.433)	(1.859)	(0.004)	(0.003)	(0.001)	(0.001)	(0.001)	(0.000)
Repeat Admin. (=1)	2.216	1.072	0.063*	0.024	−0.002	0.000	−0.003	−0.001
	(12.460)	(10.147)	(0.025)	(0.025)	(0.002)	(0.003)	(0.002)	(0.002)
Constant	−98.926	79.066	0.161	0.250*	0.022	0.027	0.024	0.019
	(114.187)	(57.523)	(0.120)	(0.117)	(0.016)	(0.030)	(0.015)	(0.012)
Observations	236	236	236	236	236	236	236	236
*R*^2^	0.0493	0.0283	0.0509	0.0204	0.0350	0.0267	0.0510	0.0323
*N* (participants)	118	118	118	118	118	118	118	118

Note: SR, sleep restricted, MES, morningness-eveningness score. BMI, body mass index. HC = high-calorie, LC = low-calorie.

*p < .05, **p < .01 for the 1-tailed test of a preregistered hypothesis (otherwise, two-tailed test *p*-value reported). Coefficient estimates shown with robust standard errors clustered on the participant in parenthesis (*n* = 2 observations per participant).

### Food liking and choice

There were also no significant effects of sleep restriction treatment, relative to a more well-rested state on food liking ratings or choice for low- or high-calorie food in study 1 or 2 (see Table 5). Robustness checks that accounted for sample selection also found no significant treatment effects (see Appendix A, Table A4).

**Table 5.** Treatment condition effects on: food liking ratings and food choice for high-calorie (HC) and low-calorie (LC) food – Study 1.

	Study 1			
	Food liking		Food choice	
Variables	HC food	LC food	HC food	LC food
SR (=1)	−0.058	−0.040	0.076	0.051
	(0.047)	(0.050)	(0.107)	(0.103)
Age	−0.055	−0.007	−0.075	0.010
	(0.032)	(0.043)	(0.062)	(0.064)
Female (=1)	0.070	0.077	−0.157	0.227
	(0.144)	(0.172)	(0.239)	(0.233)
Minority (=1)	0.021	0.163	−0.832*	0.558
	(0.308)	(0.254)	(0.394)	(0.292)
BMI	0.024	−0.009	0.021	−0.024
	(0.015)	(0.015)	(0.019)	(0.018)
MES	−0.031	−0.035	0.006	0.012
	(0.025)	(0.026)	(0.036)	(0.034)
Repeat Admin (=1)	−0.127**	−0.117*	0.144	0.153
	(0.047)	(0.050)	(0.107)	(0.103)
Constant	7.683**	7.092**	5.017**	3.655**
	(0.667)	(0.903)	(1.251)	(1.189)
Observations	236	236	236	236
*R*-squared	0.0660	0.0225	0.0497	0.0335
*N* (participants)	118	118	118	118

Note: SR, sleep restricted, MES, morningness-eveningness score; BMI, body mass index; HC, high-calorie; LC, low-calorie.

*p < .05, **p < .01 for the one-tailed test of a preregistered hypothesis (otherwise, two-tailed test *p*-value reported). Coefficient estimates shown with robust standard errors clustered on the participant in parenthesis (*n* = 2 observations per participant).

## Study 1 discussion

Study 1 findings suggest that sleep restriction, though it negatively impacts subjective (sleepiness) and objective (PVT lapses) measures, had no statistical impact on food-specific approach-avoidance biases, inhibitory control or delay discounting rates. Sleep restriction also did not have statistically significant effects on food liking or hypothetical food choices. While participant characteristics were estimated to affect some outcomes on certain tasks or on certain items of the task, there was no systematic effect of participant characteristics across tasks.

Our finding that sleep restriction did not influence approach biases for healthy or unhealthy food cues contrasts with our hypotheses that we would observe stronger approach biases for high-calorie foods, which was based on the theoretical premise of reduced deliberative processing under SR and adverse time-of-day. Future work should aim to replicate such findings using a relevant feature version of the approach-avoidance task such that participants are instructed to push/pull images in response to the content (e.g. the category of picture such as healthy or unhealthy food) rather than an irrelevant feature (e.g. the orientation of the image), which reduces demand characteristics and is more implicit in nature (Rinck & Becker, 2007). While most studies use the irrelevant feature version as it can be easily adapted for training, a meta-analysis has shown that the relevant feature version produces a stronger bias measure (Phaf et al., 2014). Indeed, more recent work also suggests the relevant feature version is required to capture attention to elicit an approach-avoidance bias (Lender et al., 2018). However, task validity in terms of predicting eating outcomes is independent of bias size and is more likely to be driven by state-dependent factors such as craving or hunger (Brockmeyer et al., 2017) – for example, animal research has also shown that approach-avoidance behaviors for food rewards differed based on hunger state and sex differences (Anversa et al., 2024).

Whilst previous work has not directly examined the influence of manipulating sleep levels on approach-avoidance biases for food, some of the previous findings have been conflicting. For example, some studies found overweight and obese individuals were faster to approach than to avoid food stimuli relative to healthy-weight individuals (Kakoschke et al., 2017a; Mehl et al., 2018), while other studies found an avoidance of food in obese individuals (Paslakis et al., 2017; Schmidt et al., 2018). Our Study 1 results found no significant impact of one’s BMI on approach-avoidance behaviors. It remains to be determined whether sleep restriction can also be classified as a ‘hot state’ that impacts the relationship between approach-avoidance bias for food and relevant outcomes like hunger or cravings, as well as potentially impacting the role of individual differences.

On a related note, the finding that there were no significant effects of sleep restriction on food liking ratings or choice for low- or high-calorie food in Study 1 is surprising, but consistent with some previous work showing that poorer sleep quality did not influence liking of low or high-calorie foods nor food choice (Pataroque, 2017). Other research has shown that partial sleep deprivation (1 day) conducted in a naturalistic setting decreased liking for low-calorie foods, but did not increase liking for high-calorie foods in healthy weight women, and also increased choice of unhealthy foods over healthy foods (Benjamins et al., 2021). In addition, other work has shown that total sleep duration increases selection of unhealthy snacks (Hogenkamp et al., 2013) and partial sleep deprivation increase liking for such snacks (McNeil et al., 2017), albeit they compared low-versus high-fat foods and only observed such effects when the task was administered in the later part of the night. Furthermore, studies have measured the relationship between sleep duration and liking found no significant relationship (Dweck et al., 2014). Future research should examine other homeostatic and non-homeostatic influences on the relationship between sleep and food liking or choice such as breakfast consumption prior to task administration (Pender et al., 2014; Stevenson et al., 2017) or food craving (Lv et al., 2018).

With regards to inhibitory control, our finding contrasts with previous studies showing that 1–2 nights of total sleep deprivation impairs performance on a general Go/No–Go task (Almklov et al., 2015; Chuah et al., 2006; Drummond et al., 2006) and food-specific version (Cedernaes et al., 2014) of the task as evidenced by increased commission errors relative to normal sleep levels. Nevertheless, our study focused on insufficient sleep rather than complete sleep deprivation. Previous studies that have examined the effect of insufficient sleep found impaired performance on a Go/No–Go task following four (Demos et al., 2016), but not one night of sleep restriction (Rossa et al., 2014). Nevertheless, these studies used neutral (i.e. letter) or emotional (i.e. faces) stimuli in the go–no/go task rather than food-specific stimuli. Studies focused on food-specific impulsivity have shown higher impulsivity in a go/no go task whereby participants were asked to respond to food-related cues after total sleep deprivation. Specifically, participants were more impulsive as indicated via a higher number of commission errors towards non-food-related cues following total sleep deprivation relative to controls, but this study lacked a condition whereby participants inhibited responses to food cues (Cedernaes et al., 2014). As such the findings cannot be clearly attributed to general or food-specific inhibitory control.

Furthermore, recent meta-analyses have indicated large effects of insufficient sleep on inhibition, albeit general rather than food-specific, as well as on tasks that captured other cognitive functions such as attention (Lowe et al., 2017). Of note, we did not observe any of the effects for approach bias on the food choice or liking tasks for the Go–No/Go task outcomes. One procedural difference between the AAT and the GNG tasks that may explain the discrepant findings is that within the AAT participants always respond whereas in the Go–No/Go task, participants respond on 75% of trials. Another difference lies in the fact that AATs usually present the cue at stimulus onset, while Go–No/Go cues appear after a given interval (e.g. 100 ms), which could stimulate participants to actively learn the link between the stimuli and response.

Delay discounting, a more general decision-making process, was also not impacted by sleep restriction. This null finding is consistent with some previous work showing that delay discounting rates did not differ between sleep restricted and well-rested participants in at-home conditions for 4 nights (Demos et al., 2016) and following one night of total sleep deprivation in the laboratory (Acheson et al., 2007; Libedinsky et al., 2013). Furthermore, others have shown that poor sleep quality impacts food but not money-related discounting rates (Law & Rasmussen, 2024). Although the results of the previous studies are consistent with that of the current study, it is possible that a longer sleep deprivation period may induce changes in delay discounting.

## Study 2: sleep restriction effects food-relevant cognitive tasks

Study 2 examined the impact of adverse time-of-day (afternoon versus middle-of the night) on inhibitory control, approach-avoidance bias and money-based delay discounting in a preregistered cross-over design experiment.

### Methods

The preregistration for Study 2 was done in a single registration on the Open Science Framework (https://doi.org/10.17605/OSF.IO/F9WXC), which covered both the protocol and other methods in one registration. The study received ethics approvals for human subjects research by the Institutional Review Board at Appalachian State University (IRB# for study approval HS-24-143), and all participants gave voluntary informed consent to participate.

### Participants and sample size

This study employed a within-subjects cross-over design for the time-of-day (TOD) manipulation. Participants were recruited from a custom list of Prolific participants who had completed a pre-screening survey on demographics and sleep habits. To approximate the characteristics of the sample collected in study 1, we recruited only U.S. participants between 18–24 years of age, who were not at significant risk of major depressive or generalized anxiety disorder, did not have extreme diurnal preferences, and did not have a self-reported or suspected sleep disorder. Instruments used to validate anxiety and depression disorder risk and diurnal preferences were the same as in Study 1. Participant sex, race, and ethnicity were available to download from the Prolific platform, but height and weight were not. Because we did not elicit height and weight during the decision task surveys for Study 2, we do not have a measure of participant BMI in Study 2.

We preregistered plans *n* = 100–120 participants for similarity to the treatment group sample size of *n* = 118 who participated in Study 1, which would leverage similar statistical power as was calculated for Study 1. Given we were unsure how many would complete the last stage of the study (i.e. the 2nd task administration that occurred approximately one week after the first task administration), we initially recruited *n* = 150 participant to complete the 1st task administration, all of whom were offered the option to complete the 2nd task administration. A final sample of *n* = 119 participants completed both parts (i.e. both times-of-day) of Study 2.

### Design and experimental manipulation

A within-subject cross-over study design was used in Study 2. Here, participants were exogenously assigned the time of day during which to complete the cognitive tasks twice, approximately one week apart, which were administered online (using the same software and interface as in Study 1). One of the task administrations required the participants to complete the task between 3pm and 5pm in the afternoon once between Tuesday and Saturday following the study being posted on Monday. Then, the following Monday, we posted the link to the second administration of the tasks to be completed between 3am and 5am during the night once between Tuesday and Saturday of that second week. The order of the Night versus Day administration was randomized during the initial sign-up survey for all participants, and though participants were given some flexibility as to the day/night during the week to complete that administration of the tasks, the protocol guaranteed at least two full days between administrations of the distinct treatment conditions. The late night-time approximates a circadian night-time that would contrast with the circadian day time administration that occurs in the afternoon. The approximation is based on previous studies using time of day to proxy the level of circadian mismatch (i.e. misalignment with one's most alert time-of-day) that one might experience at the different times of day (Dickinson et al., 2020). The time-of-day manipulation has been previously used to experimentally induce a more versus less sleepy state in a between-subjects design (Holbein et al., 2019). Here, we administered the TOD manipulation as a statistically powerful within-subjects design.

### Procedure

Prolific participants who met the eligibility criteria were randomly assigned to participate in the two-part study and randomly allocated to Day–Night or Night–Day ordering and invited to sign-up via a short survey. The main purpose of the sign-up survey was to present to participants the ordering of the two treatment conditions, which was drawn randomly at the end of the sign-up survey. Participants were also asked to input their plans for how to remember to complete both the Part 1 and Part 2 surveys, which served as a simple way to establish commitment from participants and reduce attrition. The two study parts took place approximately one week apart. For the Night condition, participants were told they would need to complete the study between 3am–5am on any day from Tuesday to Saturday of that week. They were informed that we could collect geo-location data from their survey participation for the purpose of obtaining a time-zone corrected survey start and completion time. For the Day condition, participants were asked to complete the study between 3 pm and 5 pm from Tuesday–Saturday of the week. Participants were paid a fixed fee for participating in each Part of the Study, but they were informed that the incentive bonus payments for *both* Parts 1 and 2 would only be paid if the participant completed both parts of the study within the assigned time periods.

Within the survey, participants self-reported their sleepiness on the Karolinska sleepiness scale before completing the set of AAT, GNG, MCQ, food choice, food liking, and PVT using the same software platforms as was used in Study 1. After these computerized tasks were completed, a separate decision task was administered for an unrelated study that is not relevant here. At the end of the survey (for both Parts 1 and 2) we also assessed via self-report questions to determine whether the participant had consumed caffeine in the last 2 h, and how much sleep, if any, the participant had obtained the previous night and in the last 6 h prior to testing.

### Measures

All task outcome measures were the same as in Study 1. The only difference in task methodology compared to Study 1 was that the MCQ task was purely hypothetical (i.e. non-incentivized). Recall that in Study 1 a real payoff was executed from a randomly drawn trial for a randomly drawn subset of participants. Previous research has shown that hypothetical choices in the MCQ do not differ from incentivized choices in terms of discount rates outcomes (Johnson & Bickel, 2002; Madden et al., 2003), and so we administered a hypothetical version of the MCQ for Study 2.

### Data analysis

The analysis approach was also like the approach used in Study 1 (except that we did not have height and weight measures to control for BMI in the Study 2 analysis), with the key independent measure being decision time-of-day rather than the nightly sleep level manipulation used in study 1. In Study 2, a binary *NIGHT *= 1 variable indicated the suboptimal 3am-5am time window for choices, and *NIGHT *= 0 indicated the more optimal 3pm–5pm afternoon time window for decision-making. Given that participants may have completed Part 1 of the study (i.e. the 1st task administration) but not Part 2 (i.e. the 2nd task administration approximately one week later), sensitivity analysis included a sample selection correction based on the probability of study completion, conditional on enrollment and Part 1 completion.

## Study 2 results

### Participant characteristics

The Study 2 sample had a notable higher proportion of minority participants (i.e. non-white, or white-Hispanic) compared to Study 1. Otherwise, sample characteristics of Study 2 were comparable to Study 1 in terms of age, sex, morningness-evenness measure (MES), and self-reported optimal sleep levels. Table 6 shows the sample characteristics of Study 2.

**Table 6.** Sample characteristics for Study 2.

Characteristic	Mean (SD)
Age (in years)	22.12 (1.71)
Female (%)	0.55 (.50)
Minority (%)	0.73 (.45)
MES	12.68 (3.30)
Optimal hours nightly sleep	8.01 (1.10)
Total complete participants (*n*)	119

Note: MES, morningness-eveningness score.

#### Protocol validity

**Figure 3.**
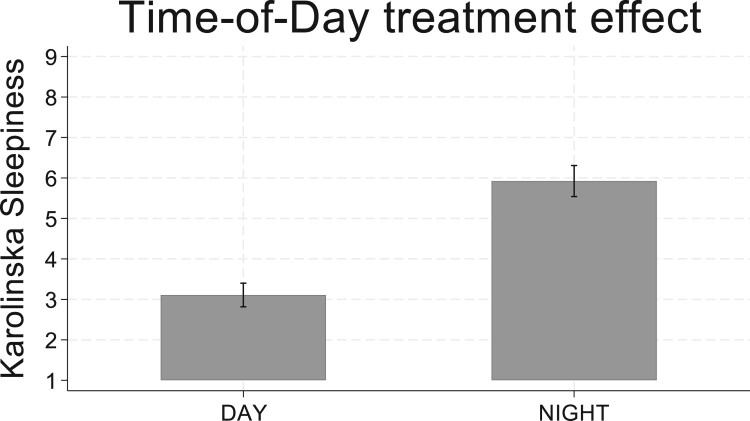
Study 2: NIGHT treatment effect on sleepiness. Mean subjective sleepiness ratings with 95% confidence interval.

Protocol validity was determined via self-reported sleepiness ratings on the KSS and objective performance on the PVT. The *NIGHT* condition produced significantly higher self-reported sleepiness ratings, relative to the afternoon condition (see Figure 3 – *Mann–Whitney non-parametric test: z* *=* *−9.194; p* *<* *.001*). For the objective PVT outcomes, the *NIGHT* condition significantly increased PVT lapses (*p* < .01; 1-tailed tests given preregistered hypotheses), but there were no treatment effects on the inverse of the mean lapse response time (see Table 7).

**Table 7.** Psychomotor vigilance task (PVT) outcomes (treatment validation) – study 2.

	Study 2	
	(1)	(2)
Variables	PVT: # lapses	PVT: Mean Lapse RT
Night (=1)	0.35**	−0.00
	(0.13)	(0.01)
Age	−0.01	−0.00
	(0.07)	(0.00)
Female (=1)	−0.11	−0.01
	(0.26)	(0.01)
Minority (=1)	−0.00	0.00
	(0.30)	(0.01)
MES	0.02	−0.00
	(0.02)	(0.00)
Repeat Admin (=1)	0.12	−0.01
	(0.14)	(0.01)
Constant	0.91	0.06
	(1.42)	(0.09)
Observations	238	164
R^2^		0.02
Chi-Squared	14.50*	

Note: MES, morningness-eveningness score.

*p < .05, **p < .01 for the one-tailed test of a preregistered hypothesis (otherwise, two-tailed test *p*-value reported). Coefficient estimates shown with robust standard errors clustered on the participant in parenthesis (*n* = 2 observations per participant). Models (1) and (3) are Poisson regressions given the dependent variable (# lapses) involves count data.

### Main preregistered hypotheses tests

The primary analyses were aimed at examining the preregistered hypotheses focused on the coefficient estimates for the NIGHT condition indicator variable in Study 2. The mean values of the outcome measures, their standard deviations, and between-condition test results with effect sizes are shown in Table 8.

**Table 8.** Study 2 outcome measures descriptive statistics.

	Day condition	Night condition	Between-condition difference test *p-value* (effect size)
*N* (participants)	119 (50.0%)	119 (50.0%)	
Approach Bias – high-calorie foods	11.697 (86.641)	−1.941 (83.271)	0.22 (0.16)
Approach Bias – low-calorie foods	−7.592 (112.467)	2.055 (77.501)	0.44 (−0.10)
Go-NoGo commission errors – high-calorie foods	0.222 (0.182)	0.241 (0.199)	0.45 (−0.10)
Go-NoGo commission errors – low-calorie foods	0.289 (0.223)	0.268 (0.230)	0.47 (0.09)
Overall discount rate (*k*-value)	0.021 (0.028)	0.022 (0.030)	0.72 (−0.05)
Small $ values discount rate (*k*-value)	0.035 (0.044)	0.037 (0.046)	0.71 (−0.05)
Medium $ values discount rate (*k*-value)	0.018 (0.025)	0.023 (0.033)	0.23 (−0.16)
Large $ values discount rate (*k*-value)	0.017 (0.031)	0.016 (0.032)	0.84 (0.03)

Notes: means shown with standard deviations in parenthesis. Approach Bias, 0 value represents no bias. For Go-NoGo, the commission errors value is the proportion of NoGo trials with an error. One-sample *t*-tests of the Approach Bias values for each treatment indicate Approach Bias scores for both high and low-calorie foods are not significantly different from zero in either the SR or WR condition (*p* > .10 in all instances for the two-tailed test). For the Between-Condition tests reported above, we ran two sample *t*-tests and report *2*-tailed *p-*values with Cohen’s *d* effect sizes in parenthesis.

### Food approach bias, commission errors, and discount rates

There were no significant effects of the *NIGHT* treatment, relative to the afternoon time-of-day, on approach-avoidance biases or commission errors for low- or high-calorie food in Study 1 (see Table 9). There was a significant effect of *NIGHT* treatment on discount rates for the medium dollar trials (*p* < .05), but not for the small or large (or pooled) dollar amount trials. The effect is such that participant choices in the *NIGHT* condition, compared to their afternoon time-of-day choices, reflect a significantly higher discount rate for that subset of medium dollar amount trials, and the estimated effect survived the sensitivity analysis that accounted for attrition-based sample selection (see Appendix A, Table A5).

**Table 9.** NIGHT treatment effects on: approach bias scores, commission errors, and delay discounting rates – study 2.

	AAT task		GNG task		MCQ task			
	Approach bias		Commission errors		Discounting rate			
Variables	HC food	LC food	HC food	LC food	All $	Small $	Med $	Large $
NIGHT (=1)	−13.744	9.898	0.019	−0.021	0.001	0.002	0.005*	−0.001
	(10.865)	(13.175)	(0.019)	(0.020)	(0.002)	(0.004)	(0.002)	(0.002)
age	5.858	1.135	−0.012	−0.008	−0.001	−0.001	−0.000	−0.002
	(3.084)	(3.331)	(0.010)	(0.012)	(0.001)	(0.002)	(0.001)	(0.002)
Female (=1)	−25.075*	−32.41**	−0.046	−0.049	−0.010*	−0.012	−0.013**	−0.008
	(10.324)	(11.169)	(0.029)	(0.036)	(0.005)	(0.007)	(0.005)	(0.005)
Minority (=1)	15.003	−13.224	0.008	0.053	0.009*	0.011	0.010**	0.008
	(11.077)	(12.846)	(0.036)	(0.040)	(0.004)	(0.007)	(0.004)	(0.004)
MES	3.629*	1.060	0.004	0.007	0.001	0.001	0.001	0.001
	(1.727)	(1.903)	(0.005)	(0.006)	(0.001)	(0.001)	(0.001)	(0.001)
Repeat Admin. (=1)	4.162	−9.939	−0.008	−0.032	0.002	0.001	−0.000	0.001
	(10.865)	(13.175)	(0.019)	(0.020)	(0.002)	(0.004)	(0.002)	(0.002)
Constant	−163.19*	−13.912	0.455	0.397	0.027	0.031	0.017	0.039
	(74.297)	(88.177)	(0.251)	(0.302)	(0.034)	(0.049)	(0.031)	(0.044)
Observations	238	238	238	238	238	238	238	238
R^2^	0.0760	0.0404	0.0310	0.0420	0.0554	0.0409	0.0877	0.0438
*N* (participants)	119	119	119	119	119	119	119	119

Note: SR, sleep restricted; MES, morningness-eveningness score; HC, high-calorie; LC, low-calorie.

*p < .05, **p < .01 for the 1-tailed test of a preregistered hypothesis (otherwise, 2-tailed test *p*-value reported). Coefficient estimates shown with robust standard errors clustered on the participant in parenthesis (*n* = 2 observations per participant).

### Food liking and choice

There were no significant effects of *NIGHT* treatment on food liking ratings or choice for low- or high-calorie food in Study 2 (see Table 10).

**Table 10.** Treatment condition effects on: food liking ratings and food choice for high-calorie (HC) and low-calorie (LC) food – study 2.

	Study 2			
	Food Liking		Food choice	
Variables	HC food	LC food	HC food	LC food
Night (=1)	−0.056	−0.086	−0.088	−0.075
	(0.054)	(0.059)	(0.092)	(0.111)
Age	0.085	0.039	0.053	−0.082
	(0.051)	(0.061)	(0.085)	(0.083)
Female (=1)	0.055	0.154	−0.501	0.566*
	(0.164)	(0.189)	(0.259)	(0.246)
Minority (=1)	0.099	0.540*	−0.392	0.397
	(0.169)	(0.236)	(0.318)	(0.312)
MES	0.034	0.020	−0.003	−0.018
	(0.023)	(0.032)	(0.040)	(0.036)
Repeat Admin (=1)	−0.062	−0.004	0.153	−0.023
	(0.054)	(0.059)	(0.092)	(0.111)
Constant	4.587**	4.594**	3.838	4.816*
	(1.212)	(1.493)	(2.012)	(1.973)
Observations	238	238	238	238
*R*^2^	0.0440	0.0613	0.0524	0.0696
*N*	119	119	119	119

Note: SR, sleep restricted; MES, morningness-eveningness score; HC, high-calorie; LC, low-calorie.

*p < .05, **p < .01 for the one-tailed test of a preregistered hypothesis (otherwise, two-tailed test *p*-value reported). Coefficient estimates shown with robust standard errors clustered on the participant in parenthesis (*n* = 2 observations per participant).

## Study 2 discussion

Study 2 findings suggest that adverse time-of-day did not significant impact any of the main outcome measures from our decision tasks (i.e. inhibitory control, approach-avoidance bias, delay discounting, food liking, and food choice).

As with Study 1, these Study 2 results did not support our preregistered hypotheses regarding adverse time-of-day effects on inhibition control and approach-avoidance bias, food liking, and food choice, and so the findings were somewhat unexpected. The theoretical premise of reduced deliberative processing under sleep restriction also motivated our hypothesis of reduced deliberative process at adverse times-of-day. Again, previous work showed that poorer sleep quality did not influence liking of low or high-calorie foods nor food choice (Pataroque, 2017), so perhaps some of our Study 2 findings are not surprising.

However, we observed a significant impact of adverse time-of-day on discount rates, such that participants in the *NIGHT* condition were less patient or future oriented for at least a certain range of monetary reward choices. This finding regarding adverse time-of-day suggests that circadian disruption may be a mechanism linking sleep to delay discounting (Baron & Culnan, 2019). This finding somewhat aligns with previous work showing that delay discounting is steeper in individuals with an evening chronotype, which suggests delay discounting may be trait-like (Milfont & Schwarzenthal, 2014). Furthermore, research has shown that circadian disruption as indicated by greater bedtime variability, but not sleep duration, was related to higher BMI in individuals with steeper delay discounting rates (Chan, 2017). Nevertheless, our study was the first to experimentally manipulate time of day and examine the effects of circadian disruption on delay discounting rates for monetary rewards. Thus, additional research is needed to examine whether circadian timing impacts a present versus future oriented bias.

### Exploratory analyses (Studies 1 and 2): moderating role of cognitive biases by treatment on food liking and choice

For both Studies 1 and 2 data, we conducted exploratory analysis to examine if one’s inhibitory control, approach-avoidance bias, or overall discount rate would moderate a link between adverse sleep state (SR or *Night* condition) and either food liking and/or food choice task outcomes. The results are reported in Table 11, with our main interest on statistically significant (*p* < .05) results that survive the robustness check that controls for sample selection, which we report in Appendix A, Table A7.

**Table 11.** Moderating role of cognitive biases by treatment condition on food liking and food choice outcome measures for Study 1 and 2.

	Study 1				Study 2			
	Food liking		Food choice		Food liking		Food choice	
Variables	LC food (1)	HC food (2)	LC food (3)	HC food (4)	LC food (5)	HC food (6)	LC food (7)	HC food (8)
SR/Night (=1)	0.021	0.033	−0.067	0.286	−0.019	−0.127	0.067	−0.114
	(0.126)	(0.121)	(0.237)	(0.267)	(0.135)	(0.109)	(0.205)	(0.205)
AAT_HC*SR/Night	−0.001	−0.001	0.001	−0.001	0.002	0.001	−0.003^	0.002^
	(0.001)	(0.001)	(0.001)	(0.001)	(0.001)	(0.001)	(0.002)	(0.001)
AAT_LC*SR/Night	0.001	0.001^	0.003^	0.000	−0.001	−0.001	0.005**	−0.003*
	(0.001)	(0.001)	(0.002)	(0.001)	(0.001)	(0.001)	(0.002)	(0.002)
CE_HC*SR/NIGHT	−0.186	−0.344	−0.067	−0.037	0.219	0.428	−0.002	0.367
	(0.413)	(0.330)	(0.708)	(0.782)	(0.526)	(0.564)	(0.805)	(0.681)
CE_LC*SR/NIGHT	0.065	−0.040	0.352	0.024	−0.291	−0.223	−0.073	−0.427
	(0.441)	(0.396)	(0.712)	(0.778)	(0.405)	(0.477)	(0.792)	(0.621)
OverallK*SR/NIGHT	−1.211	−0.418	1.054	−10.062*	−1.106	1.044	−2.334	0.796
	(2.587)	(1.894)	(4.614)	(5.117)	(1.972)	(1.722)	(5.220)	(4.604)
AAT_HC	0.001	0.001	−0.001	−0.001	0.000	0.000	0.002	−0.001
	(0.001)	(0.001)	(0.001)	(0.001)	(0.001)	(0.000)	(0.002)	(0.001)
AAT_LC	−0.001	−0.001	−0.001	−0.001	−0.000	0.001	−0.004**	0.003**
	(0.001)	(0.001)	(0.001)	(0.001)	(0.000)	(0.000)	(0.001)	(0.001)
CE_HC	0.156	−0.221	−0.162	0.439	−0.120	0.098	−0.160	−0.412
	(0.257)	(0.258)	(0.482)	(0.539)	(0.354)	(0.429)	(0.651)	(0.537)
CE_LC	0.187	0.262	−0.121	0.039	0.219	−0.132	0.581	−0.032
	(0.314)	(0.275)	(0.529)	(0.591)	(0.351)	(0.373)	(0.466)	(0.439)
Overall_K	0.159	0.559	4.244	2.700	1.599	4.378*	−8.796*	4.675
	(2.748)	(2.417)	(4.587)	(4.371)	(2.111)	(2.135)	(3.912)	(3.801)
Observations	236	236	236	236	238	238	238	238
R-squared	0.0192	0.0658	0.0506	0.0614	0.084	0.096	0.108	0.087

Note: SR, Sleep Restricted. HC, high-calorie, LC, low-calorie. AAT_HC and AAT_LC refer to approach-avoidance bias scores for high-calorie and low-calorie foods, respectively. CE_HC and CE_LC refer to GNG commission errors for high-calorie and low-calorie foods, respectively. Overall_K refers to the discount rate estimate from all Monetary Choice Questionnaire task trials.

**p < 0.01, *p < 0.05, ^*p* < .10. Coefficient estimates shown with robust standard errors clustered on the participant in parenthesis (*n* = 2 observations per participant). All models included controls for participant characteristics (age, sex, minority status, chronotype), session controls, and BMI for Study 1 (suppressed for space considerations).

### Food liking

In both Studies 1 and 2, there were no significant cognitive moderating effects observed for food liking (i.e. the OverallK*SR/NIGHT interaction term). In Study 2, there was a significant main effect of *Overall_K* with high-calorie item food liking ratings. The positive and significant coefficient estimate (*p* < .05) indicated that steeper discounting rates predicted higher liking ratings for high-calorie food items regardless of time of day (model (6)), but this effect did not survive the robustness check that accounted for sample selection with the IPW correction (Appendix Table A7, model (6)). The latter means that the significant results are due primarily to sample selection such that those participants who completed the study were different than those who dropped out.

### Food choice

In Study 1, *OverallK* was estimated to moderate an effect of SR lowering one’s number of high-calorie foods chosen in the food choice task, which is surprising. However, this effect did not survive the sensitivity analysis (i.e. robustness check in Appendix Table A7). There were no significant interactions between SR and AAT or GNG measures in predicting food choice outcomes in Study 1.

In Study 2, an intuitive negative main effect of *Overall_K* (*p* < .05) indicated that higher discount rates predicted fewer low-calorie food choices, but this effect was not robust to the sensitivity analysis we conducted in Appendix A, Table A7. There was no significant interaction between *Overall_K* and *NIGHT* condition. There were also no significant main effects of GNG commission errors or interaction effects between commission errors for low- or high-calorie foods and *NIGHT* condition. There were also no significant main or interaction effects with *NIGHT* for approach bias for high-calorie food choices.

However, the Study 2 data indicated that an approach-avoidance bias for low-calorie food choices moderated a *NIGHT* condition effect on both high-calorie and low-calorie food choices. In columns (7) and (8) of Table 11 we observe that a main effect indicated that a stronger approach bias for low-calorie food (i.e. the AAT_LC coefficient estimate) predicted fewer low-calorie food choices and more high-calorie food choices for the reference-group (*Night = 0* condition). However, these effects were mitigated when examining the low-calorie food approach bias interaction with *NIGHT* condition. That is, the combined main and interaction effects for (*AAT_LC* + AAT_LC)*NIGHT were statistically no different from zero (*p* > .10) on the *F*-test of the linear coefficient combinations in columns (7) and (8) of Table 11. Importantly, these effects passed a robustness check and remained significant when controlling for attrition (sample selection) in the comparable IPW-corrected models (Appendix Table A7, columns (7) and (8)).

**Figure 4.**
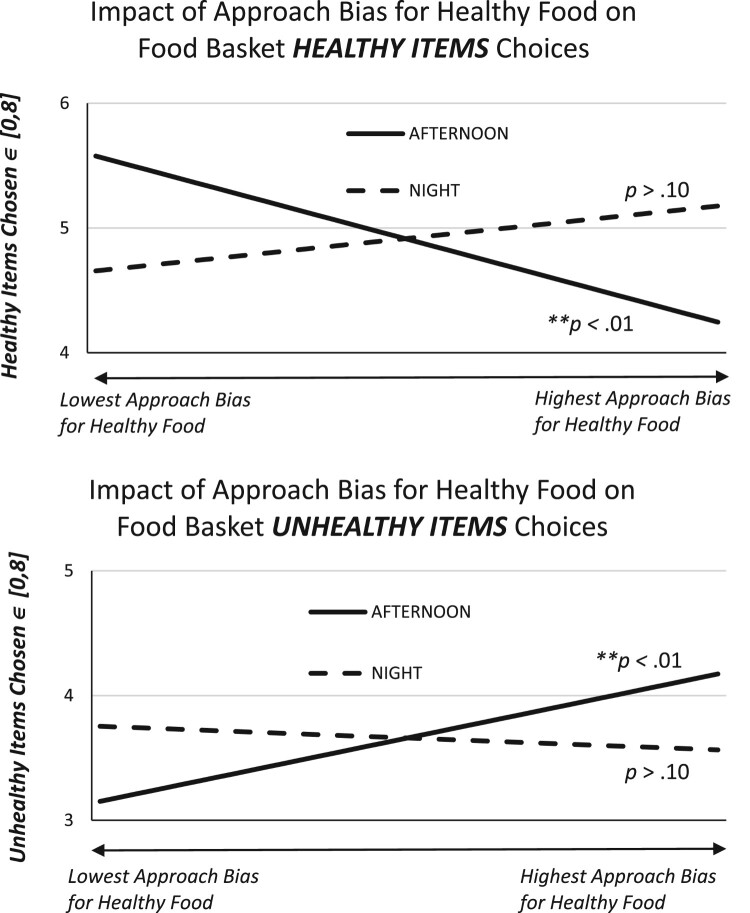
Forecast are derived from Table 11 estimates, which are plotted using the range of healthy food approach bias values (*x*-axis) from −184 to +186 (the approximate 95% center range of observed values in the Study 2 sample). The y-axis is the predicted number of *Healthy* (or ‘low-calorie', top panel) or *Unhealthy* (‘high-calorie', bottom panel) food items chosen from a maximum possible 8 items to choose in the task. The range of *y*-axis values are truncated in the above figures to focus on the predicted range of choices.

To help visualize these significant interaction effects for the Study 2 Food Choice models, Figure 4 plots the forecasted effect of one’s *AAT_LC* approach bias. The predicted number of LC or HC food choices is plotted using the constant term and significant coefficient estimates from the Table 11 results for Study 2, and we plot these forecasts for the range of *AAT*_*LC* bias values observed in our sample (removing the 2.5% extreme-value tails).

## General Discussion

We conducted two experimental studies designed to examine within-subjects manipulations of sleep restriction (Study 1) and time-of-day (Study 2) on food-specific and general decision-making as well as food liking and choice. While both exogenously assigned adverse sleep states increased sleepiness ratings and lapses on the PVT as expected, these sleep states did not significantly affect most of the cognitive decision-related outcomes we examined. The only support found for our preregistered hypotheses was a robust find that *NIGHT* condition (Study 2) predicted a significantly higher money choice discount rate. Even then, this result was restricted to the subset of trials that involved medium-sized monetary amounts.

Exploratory analysis revealed a robust moderating effect of *NIGHT* condition on the link between a low-calorie food approach bias predicting fewer low-calorie and more high-calorie food choices in a hypothetical time-constrained food choice task. Both the main and the moderating effects are somewhat counterintuitive here. First, the main effect suggests a tendency to select more unhealthy, and fewer healthy, food basket items in a well-rested or afternoon time-of-day condition (i.e. more optimal sleep states) when one displays a bias *towards* healthier low-calorie food items. And, the moderating effect suggests that the more adverse sleep state, which generates sleepier decision-making, eliminates this bias such that approach bias towards healthier food items no longer significantly impacts one’s food choices. If one believes that sleepy decisions are more automatic (and less deliberative) decisions (Dickinson, 2021), then one would anticipate a stronger link between a bias towards a category of food and the selection of that same category of food during the *NIGHT* condition. We did not find this, although *NIGHT* condition did eliminate a counterintuitive link between the bias and revealed preference towards the opposing food category. This exploratory finding, therefore, may not align with the notion that approach-avoidance bias results from more implicit or automatic thinking (see arguments in Meule et al., 2019). Clearly, a better understanding of how adverse time-of-day may moderate a food-specific decision bias effect on food choices warrants additional research.

### Strengths, limitations and future directions

The findings demonstrated here are relatively robust given they were demonstrated across two separate experimental studies. The naturalistic sleep protocol used generated SR levels typical in real-world environments, and we increased statistical power using a within-subject sleep manipulation. We also verified the experimental manipulation of sleep via objective, but passive measurements of sleep levels. This protocol strikes a balance between the highly controlled sleep lab study that manipulates sleep (often to extreme levels) in a foreign environment and observational studies that lack experimental control necessary to identify causation. As a result, our findings promise to have broad impacts that can apply quite directly to the public.

Nevertheless, the study is also subject to several limitations. We recruited a college student sample comprising mostly young adults who may have later bedtimes. Therefore, the findings may not generalize to other populations such as middle or older adults given that sleep changes with ageing. Specifically, cognitive performance is better maintained following prolonged wakefulness in older people, particularly for performance accuracy, which declines more in younger people (Alhola & Polo-Kantola, 2007). Furthermore, food-related reward signals may be heightened under sleep restriction more generally in adolescents whose inhibitory control processes are less developed (Simon et al., 2015). Future research will help identify the importance of brain development in determining the impact of insufficient sleep on food choice and other related behaviors. We also did not assess neural activity during the cognitive nor food choice tasks in the current study, which may shed important insights given previous work showing that baseline ventral striatum responsivity moderates the link between sleep restriction and dietary choice (Satterfield et al., 2019).

The use of hypothetical rewards compared to real rewards in for the MCQ across our two studies may be considered a limitation given the change in methodology, but our implementation of a within-subjects design somewhat mitigates this concern as the key comparison for an adverse sleep behavioral change is made with respect to one’s own baseline in the preferred sleep state or time-of-day. Another difference between Studies 1 and 2 is that we did not have a measure of participant BMI in Study 2. Thus, while estimates on the BMI control variable were generally statistically insignificant in Study 1, it would be useful to include a similar control measure in the Study 2 estimations.

Furthermore, it would be useful to examine the impact of other sleep-related factors on cognitive performance given previous work showing that night-to-night variability impacts decision outcomes such as monetary risk tasking (Dickinson et al., 2022). Finally, we measured hypothetical food choice in the food images choice task, rather than actual food choices (i.e. providing the selected snack) and we did not measure food intake such as during an ad libitum taste test, which may be more comparable to real-world dietary decisions. Taken together, our main findings provide evidence that sleepiness does not impact food-related choices and cognition as hypothesized, but exploratory findings suggest potentially important moderating effects. Further investigation is warranted to replicate such findings and clarify any important links between sleepiness and its impact on food-related decision-making.

## Data Availability

The data that support the findings of this study are openly available on the Open Science Framework. The project locator is DOI: 10.17605/OSF.IO/P2TEQ, and the data are in ‘Sleep Restriction Time-of-Day and Food-GNG Food-AAT and Delay Discounting’ folder under the Files tab for the project. The data are also available from the authors on request (contact: David Dickinson at dickinsondl@appstate.edu).

## Appendix

**Table A1.** Sample selection equations (used to generate inverse probability weights).

	Study 1	Study 2
Variables	Finished	Finished
Female ( = 1)	0.30	−0.05
	(0.30)	(0.16)
Minority/Race ( = 1)	−0.08	0.56**
	(0.41)	(0.17)
Age (years)	0.06	0.03
	(0.07)	(0.05)
College Student ( = 1)		−0.13
		(0.17)
Self-reported nightly sleep last week (h/night)	−0.14	
	(0.18)	
Self-reported nightly sleep last night (h)	−0.02	
	(0.11)	
Self-perceived optimal nightly sleep (h/night)	0.24	−0.11
	(0.16)	(0.07)
Depression (PHQ-2 risk score)	−0.12	0.15
	(0.16)	(0.11)
Anxiety (GAD-7 risk score)	0.02	−0.04
	(0.06)	(0.03)
Epworth daytime sleepiness	−0.12**	−0.03
	(0.05)	(0.02)
Morningness-eveningness score	0.02	−0.01
	(0.05)	(0.02)
Control condition participant	0.58	
	(0.42)	
Study 2 NIGHT condition first ( = 1)		−0.09
		(0.16)
Constant	0.27	0.24
	(2.76)	(1.30)
Observations	175	268
Chi-squared	15.30	19.69
*R*-squared	0.123	0.0533

Standard errors in parentheses. ***p* < 0.01, **p* < .05.

**Study 1**: *n* = 176 participants enrolled (*n* = 155 completed) study. All were college students. One participant omitted due to incomplete data from online screening survey. *Race* as a category used in place of *Minority* given the latter predicted completing the study (i.e. the few *Minority *= 1 participants all completed Study 1).

**Study 2**: *n* = 268 participants enrolled (*n* = 122 completed) study from the Prolific participant pool (3 participants experienced software errors on the decision tasks, which made their decision data unusable). Results do not differ is using the same set of regressors as in the Study 1 model (other than *Control*, which did not exist for Study 2). Only *Minority* status predicts Study 2 completion.

**Table A2.** SR/NIGHT condition effects on PVT outcomes (treatment validation) – study 1 and study 2. (Robustness check: Inverse Probability Weight sample selection correction)

	Study 1		Study 2	
	(1)	(2)	(3)	(4)
Variables	PVT: # lapses	PVT: Mean Lapse RT	PVT: # lapses	PVT: Mean Lapse RT
SR ( = 1)	0.296*	−0.023		
	(0.174)	(0.024)		
Night ( = 1)			0.34*	−0.00
			(0.14)	(0.01)
Age	−0.088	−0.000	−0.01	0.00
	(0.100)	(0.005)	(0.06)	(0.00)
Female ( = 1)	−0.314	−0.005	−0.06	−0.01
	(0.356)	(0.030)	(0.24)	(0.01)
Minority ( = 1)	−0.145	−0.014	0.09	0.00
	(0.334)	(0.024)	(0.26)	(0.01)
BMI	0.033*	0.000		
	(0.015)	(0.001)		
MES	−0.005	−0.000	0.01	−0.00
	(0.044)	(0.002)	(0.03)	(0.00)
Repeat Admin ( = 1)	0.435**	0.034	0.13	−0.01
	(0.149)	(0.020)	(0.14)	(0.01)
Constant	1.011	0.037	0.85	0.03
	(1.999)	(0.132)	(1.40)	(0.07)
Observations	234	121	238	164
R^2^		0.032		0.02
Chi-Squared	26.49**		11.15*	

Note: SR = sleep restricted. PVT, psychomotor vigilance task. BMI, body mass index. MES, morningness-eveningness score. RT = response time.

**p* < .05, ***p* < .01 for the 1-tailed test of a preregistered hypothesis (otherwise, 2-tailed test *p*-value reported). Robust standard errors clustered on the participant (*n* = 2 observations per participant). Models (1) and (3) are Poisson regressions given the dependent variable (# lapses) involves count data. All models are weighted regressions that use the inverse probability weight correction derived from a sample selection model in Table A1, which estimates the probability of completing the study (conditional on enrollment).

**Table A3.** Treatment condition effects on: approach bias scores, commission errors, and delay discounting rates – study 1 (robustness check: Inverse Probability Weight sample selection correction).

	AAT task		GNG task		MCQ task			
	Approach bias		Commission errors		Discounting rate			
Variables	HC food	LC food	HC food	LC food	All $	Small $	Med $	Large $
SR ( = 1)	14.340	2.703	−0.042*	−0.038	0.001	0.003	−0.001	0.001
	(12.492)	(10.083)	(0.025)	(0.026)	(0.002)	(0.003)	(0.002)	(0.002)
Age	7.492	−3.656	0.002	0.000	−0.001	−0.001	−0.001	−0.001
	(7.329)	(3.452)	(0.005)	(0.006)	(0.001)	(0.001)	(0.001)	(0.001)
Female ( = 1)	5.908	6.419	−0.032	0.005	−0.007	−0.009	−0.009*	−0.005
	(13.363)	(10.017)	(0.026)	(0.024)	(0.004)	(0.007)	(0.003)	(0.003)
Minority ( = 1)	56.566	23.025*	−0.033	0.035	−0.001	0.007	−0.003	−0.001
	(28.694)	(9.972)	(0.046)	(0.038)	(0.004)	(0.010)	(0.003)	(0.003)
BMI	−0.861	0.497	0.001	0.000	0.000	0.000	0.000	0.000
	(1.005)	(0.884)	(0.002)	(0.002)	(0.000)	(0.001)	(0.000)	(0.000)
MES	−2.903	−1.715	0.001	0.002	0.000	0.001	0.001	0.000
	(2.434)	(1.792)	(0.004)	(0.003)	(0.001)	(0.001)	(0.001)	(0.000)
Repeat Admin. ( = 1)	2.286	2.040	0.064*	0.022	−0.002	0.000	−0.003	−0.001
	(12.492)	(10.083)	(0.025)	(0.026)	(0.002)	(0.003)	(0.002)	(0.002)
Constant	−100.341	76.434	0.187	0.251*	0.022	0.028	0.024	0.019
	(116.018)	(57.648)	(0.123)	(0.118)	(0.016)	(0.029)	(0.015)	(0.012)
Observations	236	236	236	236	236	236	236	236
R^2^	0.049	0.030	0.051	0.019	0.037	0.027	0.054	0.033
*N*	118	118	118	118	118	118	118	118

Note: SR = sleep restricted. BMI, body mass index. MES, morningness-eveningness score.

**p* < .05, ***p* < .01 for the 1-tailed test of a preregistered hypothesis (otherwise, 2-tailed test *p*-value reported). Robust standard errors clustered on the participant (*n* = 2 observations per participant). All models are weighted regressions that use the inverse probability weight correction derived from a sample selection model in Table A1, which estimates the probability of completing the study (conditional on enrollment).

**Table A4.** Treatment condition effects on: food liking ratings and food choice for high-calorie (HC) and low-calorie (LC) food – study 1 (Robustness check: Inverse Probability Weight sample selection correction).

	Study 1			
	Food liking		Food choice	
Variables	HC food	LC food	HC food	LC food
SR ( = 1)	−0.058	−0.040	0.076	0.051
	(0.047)	(0.050)	(0.107)	(0.103)
age	−0.055	−0.007	−0.075	0.010
	(0.032)	(0.043)	(0.062)	(0.064)
Female ( = 1)	0.070	0.077	−0.157	0.227
	(0.144)	(0.172)	(0.239)	(0.233)
Minority ( = 1)	0.021	0.163	−0.832*	0.558
	(0.308)	(0.254)	(0.394)	(0.292)
BMI	0.024	−0.009	0.021	−0.024
	(0.015)	(0.015)	(0.019)	(0.018)
MES	−0.031	−0.035	0.006	0.012
	(0.025)	(0.026)	(0.036)	(0.034)
Repeat Admin ( = 1)	−0.127**	−0.117*	0.144	0.153
	(0.047)	(0.050)	(0.107)	(0.103)
Constant	7.683**	7.092**	5.017**	3.655**
	(0.667)	(0.903)	(1.251)	(1.189)
Observations	236	236	236	236
*R*-squared	0.0660	0.0225	0.0497	0.0335
*N* (participants)	118	118	118	118

Note: SR, sleep restricted; BMI, body mass index; MES, morningness-eveningness score; HC,  high-calorie; LC, low-calorie.

**p* < .05, ***p* < .01 for the one-tailed test of a preregistered hypothesis (otherwise, 2-tailed test *p*-value reported). Robust standard errors clustered on the participant (*n* = 2 observations per participant). All models are weighted regressions that use the inverse probability weight correction derived from a sample selection model in Table A1, which estimates the probability of completing the study (conditional on enrollment).

**Table A5.** Treatment condition effects on: approach bias scores, commission errors, and delay discounting rates – study 2 (robustness check: Inverse Probability Weight sample selection correction)

	AAT task		GNG task		MCQ task			
	Approach bias		Commission errors		Discounting rate			
Variables	HC food	LC food	HC food	LC food	All $	Small $	Med $	Large $
NIGHT ( = 1)	−14.167	5.869	0.017	−0.024	0.002	0.004	0.004*	0.000
	(10.507)	(14.377)	(0.019)	(0.021)	(0.002)	(0.004)	(0.002)	(0.002)
age	7.814*	2.156	−0.011	−0.006	−0.001	−0.001	−0.000	−0.002
	(3.146)	(3.642)	(0.012)	(0.013)	(0.001)	(0.002)	(0.001)	(0.002)
Female ( = 1)	−26.757**	−26.673*	−0.041	−0.036	−0.010*	−0.012	−0.013**	−0.008
	(9.840)	(10.536)	(0.032)	(0.037)	(0.005)	(0.008)	(0.005)	(0.005)
Minority ( = 1)	14.641	−13.429	0.016	0.057	0.008	0.011	0.010*	0.008
	(10.560)	(12.388)	(0.038)	(0.042)	(0.004)	(0.007)	(0.004)	(0.004)
MES	3.424*	0.572	0.001	0.004	0.001	0.001	0.000	0.001
	(1.708)	(1.876)	(0.005)	(0.006)	(0.001)	(0.001)	(0.001)	(0.001)
Repeat Admin. ( = 1)	1.363	−14.439	−0.011	−0.028	0.001	0.001	−0.001	0.000
	(10.507)	(14.377)	(0.019)	(0.021)	(0.002)	(0.004)	(0.002)	(0.002)
Constant	−201.81**	−28.298	0.471	0.370	0.034	0.049	0.019	0.038
	(75.96)	(101.343)	(0.306)	(0.343)	(0.031)	(0.049)	(0.027)	(0.038)
Observations	238	238	238	238	238	238	238	238
R^2^	0.093	0.034	0.024	0.033	0.063	0.043	0.092	0.050
*N* (participants)	119	119	119	119	119	119	119	119

Note: MES, morningness-eveningness score. HC = high-calorie. LC = low-calorie.

**p* < .05, ***p* < .01 for the 1-tailed test of a preregistered hypothesis (otherwise, 2-tailed test *p*-value reported). Robust standard errors clustered on the participant (*n* = 2 observations per participant).

**Table A6.** Treatment condition effects on: food liking ratings and food choice for high-calorie (HC) and low-calorie (LC) food – Study 2 (Robustness check: Inverse Probability Weight sample selection correction).

	Study 1			
	Food liking		Food choice	
Variables	HC food	LC food	HC food	LC food
NIGHT ( = 1)	−0.064	−0.107	−0.139	−0.018
	(0.049)	(0.064)	(0.095)	(0.103)
Age	0.092	0.068	0.007	−0.042
	(0.050)	(0.068)	(0.088)	(0.086)
Female ( = 1)	0.024	0.054	−0.486	0.568*
	(0.159)	(0.204)	(0.278)	(0.265)
Minority ( = 1)	0.105	0.545*	−0.458	0.461
	(0.167)	(0.236)	(0.317)	(0.309)
MES	0.034	0.012	0.006	−0.024
	(0.023)	(0.035)	(0.043)	(0.039)
Repeat Admin ( = 1)	−0.067	0.001	0.126	0.005
	(0.049)	(0.064)	(0.095)	(0.103)
Constant	4.457**	4.119*	4.854*	3.900
	(1.178)	(1.730)	(2.139)	(2.083)
Observations	238	238	238	238
R-squared	0.053	0.069	0.053	0.070
*N* (participants)	119	119	119	119

Note: MES, morningness-eveningness score. HC, high-calorie. LC, low-calorie.

**p* < .05, ***p* < .01 for the 1-tailed test of a preregistered hypothesis (otherwise, 2-tailed test *p*-value reported). Robust standard errors clustered on the participant (*n* = 2 observations per participant). All models are weighted regressions that use the inverse probability weight correction derived from a sample selection model in Table A1, which estimates the probability of completing the study (conditional on enrollment).

**Table A7.** Moderating role of cognitive biases by condition on Food Liking ratings by study (robustness check: inverse probability weight sample selection correction). Moderating role of cognitive biases by treatment condition on food liking and choice for Study 1 and 2.

	Study 1				Study 2			
	Food liking		Food choice		Food liking		Food choice	
Variables	LC food	HC food	LC food	HC food	LC food	HC food	LC food	HC food
SR/Night ( = 1)	−0.278	−0.318^	−0.241	0.317	−0.123	−0.361**	0.131	−0.227
	(0.279)	(0.187)	(0.373)	(0.363)	(0.182)	(0.136)	(0.237)	(0.265)
AAT_HC*SR/Night	−0.000	−0.000	0.000	−0.001	0.002	0.001	−0.001	0.000
	(0.001)	(0.001)	(0.002)	(0.002)	(0.002)	(0.001)	(0.002)	(0.002)
AAT_LC*SR/Night	−0.002	0.001	0.001	0.002	0.002	−0.003*	0.006*	−0.006*
	(0.002)	(0.002)	(0.002)	(0.002)	(0.002)	(0.001)	(0.002)	(0.003)
CE_HC*SR/NIGHT	0.992	0.257	0.770	−1.154	0.179	0.433	0.675	−0.002
	(0.683)	(0.578)	(0.989)	(1.040)	(0.855)	(0.823)	(1.347)	(1.355)
CE_LC*SR/NIGHT	0.362	0.477	0.569	0.370	0.161	0.466	−0.618	−0.042
	(0.964)	(0.673)	(1.082)	(1.186)	(0.724)	(0.714)	(1.112)	(1.158)
OverallK*SR/NIGHT	−5.493	2.190	−3.236	−2.964	−1.853	2.362	−3.450	2.490
	(3.681)	(3.381)	(5.837)	(6.058)	(2.896)	(2.452)	(5.391)	(5.186)
AAT_HC	−0.000	0.000	−0.001	0.000	0.001	0.000	0.002	−0.002
	(0.001)	(0.001)	(0.001)	(0.002)	(0.001)	(0.001)	(0.002)	(0.002)
AAT_LC	0.001	−0.002	0.002	−0.004*	−0.002	0.002*	−0.004**	0.004*
	(0.002)	(0.001)	(0.002)	(0.002)	(0.001)	(0.001)	(0.001)	(0.002)
CE_HC	−0.107	−0.571	−0.491	0.840	−0.011	0.180	0.018	−0.601
	(0.416)	(0.488)	(0.633)	(0.687)	(0.616)	(0.631)	(0.977)	(0.956)
CE_LC	0.248	−0.256	0.489	−0.763	−0.140	−0.598	1.049	−0.494
	(0.612)	(0.449)	(0.788)	(0.828)	(0.582)	(0.531)	(0.827)	(0.878)
Overall_K	3.850	−5.083	7.526	−2.245	1.257	3.759	−4.700	3.360
	(3.520)	(3.685)	(6.423)	(5.864)	(2.946)	(2.736)	(4.476)	(5.082)
Observations	236	236	238	238	238	238	238	238
*R*-squared	0.064	0.096	0.078	0.089	0.116	0.100	0.145	0.114

Note: SR, Sleep Restricted. HC, high-calorie, LC, low-calorie. AAT_HC and AAT_LC refer to approach-avoidance bias scores for high-calorie and low-calorie foods, respectively. CE_HC and CE_LC refer to GNG commission errors for high-calorie and low-calorie foods, respectively. Overall_K refers to the discount rate estimate from all Monetary Choice Questionnaire task trials.

***p* < 0.01, **p* < 0.05, ^*p* < .10. Robust standard errors in parenthesis. Models are random effects generalized least squared estimates that account for multiple observations per participant (*n* = 2). All models included controls for participant characteristics (age, sex, minority status, chronotype), session control, and BMI for Study 1 (suppressed for space considerations).
